# Nitrogen deposition significantly alters the nonadditive effects of mixed litter decomposition on soil bio and chemical properties

**DOI:** 10.3389/fpls.2026.1865577

**Published:** 2026-07-07

**Authors:** Liping Li, Yangfei Zhang, Xiaoxi Zhang, Wenqing Jiang, Jing Xie, Xianyang Pan, Kaixuan Liu, Xin Liu, Xincheng Huang

**Affiliations:** 1 College of Life Sciences, Yan’an University, Yan’an, Shaanxi, China; 2 Key Laboratory for Applied Ecology of Loess Plateau, Yan’an, Shaanxi, China; 3 Baoji Agro-Tech Extension and Service Center, Baoji, Shaanxi, China

**Keywords:** mixed litter decomposition, nitrogen deposition, nonadditive effects, species and chemical diversity, species composition, subsequent ecological effects

## Abstract

**Introduction:**

Nitrogen deposition has been confirmed to significantly affect mixed decomposition of litter. However, it remains unclear whether it alters the nonadditive effects (NAEs) of this process on soil bio and chemical properties and whether such alterations are mediated by the diversity and species composition of litter mixtures.

**Methods:**

In this study, a decomposition experiment was conducted with seven litter mixtures composed of four dominant plant species in *Robinia pseudoacacia* plantations in the Loess Hilly Region, China, under nitrogen deposition treatments ranging from 0 to 12 g·m^-2^·yr^-1^. The NAEs of mixed litter decomposition on soil properties, the changes in these NAEs with increasing nitrogen deposition rates (CNAEs), and the responses of the CNAEs to alterations in the diversity (chemical diversity and species diversity) and species composition of litter mixtures were investigated.

**Results:**

The main results are as follows: (1) Generally, nitrogen deposition altered the NAEs of mixed litter on soil organic carbon and available phosphorus replenishment toward synergistic effects (including weakening antagonistic effects, enhancing synergistic effects, and their intermediate states) and shifted its effects on soil available nitrogen replenishment toward antagonistic effects, whereas no consistent pattern was observed in the alterations of its effects on soil acidification. (2) Nitrogen deposition shifted the NAEs of mixed litter on stimulating soil sucrase and urease activities toward antagonistic and synergistic effects, respectively. Its effects on catalase activity shifted toward antagonism under low nitrogen deposition rates but remained additive under high nitrogen deposition rates. In contrast, no consistent regulatory pattern was detected for its effects on alkaline phosphatase activity. (3) Under the conditions of high chemical diversity (richness and dispersion) of mixed litter and the presence of *R. pseudoacacia* litter, the NAEs on the efficacy of supplementing soil carbon and nitrogen nutrients and stimulating the activities of soil sucrase, phosphatase and urease tended to show as synergistic effects under high-level nitrogen deposition; meanwhile, the NAEs on the efficacy of stimulating soil catalase activity and acidifying the soil tended to show antagonistic effects. However, the increase in the number of species in the mixture and the presence of *Chrysanthemum indicum* and *Artemisia gmelinii* litter produce the opposite effects.

**Conclusion:**

Nitrogen deposition alters the nonadditive effects of mixed litter decomposition on soil properties and enzyme activities, with these changes regulated by litter diversity and species composition.

## 1 Introduction

Since the Industrial Revolution, atmospheric nitrogen deposition caused by anthropogenic activities has far exceeded its natural level. Over the past 30 years, the average nitrogen deposition rate in China has rapidly increased from 1.32 g·m^-2^·yr^-1^ to 2.11 g·m^-2^·yr^-1^ (Yang et al., 2017), making China one of the regions with the highest nitrogen deposition. Moreover, the influence of nitrogen deposition has been continuously expanding to the inland areas of northwest China, along with a significant increase in deposition amount (Zhang et al., 2021).

Previous studies have shown that excessive nitrogen input not only induces changes in the physical, chemical and biological properties of soil, as well as the growth, physiological and community characteristics of plants but also significantly affects the interactions between plants and soil and the ecosystem functions governed by such interactions (Chen et al., 2020; Zhang et al., 2021). Litter decomposition is one of the core processes of material cycling and energy flow in ecosystems and plays a crucial role in maintaining soil fertility and vegetation regeneration (Chen et al., 2017; Otaki and Tsuyuzaki, 2019). A thorough understanding of the effects of nitrogen deposition on this key ecological process is of great significance for clarifying its potential long-term disturbances to ecosystems. However, most current studies have focused more on the effects and mechanisms of nitrogen deposition on litter decomposition itself (Hou et al., 2018; Pichon et al., 2020), whereas few have reported whether it affects the subsequent ecological effects of litter decomposition—such as its impacts on soil chemical and biological properties.

Notably, litter from different plants generally decomposes together in natural ecosystems. Such mixed decomposition often obviously improves the microenvironment within the litter layer (including spatial structure, and moisture and aeration conditions), provides more balanced and complementary nutrients for decomposers, induces nutrient transfer among litters to enhance their overall decomposability, and alters the concentration distribution of decomposition inhibitors (e.g., phenolic secondary metabolites) in the litter layer, thereby affecting the growth and activity of decomposers and, consequently, the litter decomposition processes (Frainer et al., 2015; Gartner and Cardon, 2004).

Accordingly, mixed litter decomposition in most cases induces significant nonadditive effects (NAEs) (Hu and Yang, 2020; Kou et al., 2020; Li et al., 2016b; Zhang et al., 2019b), which profoundly influence nutrient return and metabolite input from litter to soil. Correspondingly, mixed litter decomposition usually causes NAEs in soil biochemical properties. For instance, Zhou et al (Zhou et al., 2023). reported that the soil nitrate nitrogen content was significantly lower than expected under treatments of *Lespedeza davurica*, *Artemisia gmelinii* and *A. annua* mixed litter. Li et al (Li et al., 2020). found that mixed decomposition of *R. pseudoacacia* litter with *Larix principis-rupprechtii* or *Betula platyphylla* litter synergistically stimulated the activities of most soil enzymes, whereas its mixture with *Pinus tabuliformis* litter synergistically increased soil fungal abundance. Considering that this phenomenon is one of the key factors regulating the subsequent ecological effects of litter decomposition, exploring its response mechanisms to nitrogen deposition is of great theoretical significance for further understanding the potential disturbances of nitrogen deposition to ecosystems.

Essentially, the NAEs of mixed litter decomposition on soil properties are achieved by altering the release rates of litter nutrients and organic components through mixed decomposition effects. Recent studies have indicated that exogenous nitrogen input may significantly modify the NAEs on litter decomposition by either reducing nutrient gradients among litters and inhibiting fungal mycelial growth (Shen et al., 2019; Zhang et al., 2018) (thus constraining nutrient transfer between litters) or altering the unique microbial community structure and functions of mixed litters by influencing the growth of specific microorganisms (Entwistle et al., 2018; Morrison et al., 2018; Shen et al., 2019; Zhang et al., 2018). Consequently, nitrogen deposition is highly likely to change the NAEs of mixed litter decomposition on soil properties. However, due to the limitations of existing studies, the above hypothesis remains untested. In addition, considering that the occurrence, direction and intensity of NAEs are often related to the diversity of mixed litter (including species and chemical diversity) and the presence or absence of specific species (Chen et al., 2017; Li et al., 2023; Lin et al., 2021), whether there are differences in the NAEs of mixed decomposition of litter with different diversity on soil properties under nitrogen deposition remains to be verified.

Soils in the Chinese Loess Plateau are infertile, and 60–90% of the nutrients required for vegetation growth are obtained from litter decomposition and nutrient return. Studies have shown that during the mixed decomposition of litter from 13 local plantation tree species, approximately 60% of the combinations can significantly synergistically promote nitrogen and phosphorus release of litter, with the maximum increment reaching up to 63% (Zhu, 2012). In addition, this effect is being negatively interfered with by increasing nitrogen deposition (Li et al., 2025, Li et al., 2026). However, it remains unclear whether the above phenomena will ultimately alter the soil ecological effects of mixed litter decomposition. Therefore, this study took the litter of four plant species, namely *R. pseudoacacia*, *A. gmelinii*, *C. indicum*, and *Stipa grandis*, from the main local plantation—*R. pseudoacacia* plantation—and seven mixed litters composed of them as research objects. Conducting a laboratory-simulated decomposition incubation experiment under nitrogen addition treatments at rates of 0, 4, 8, and 12 g·m^-2^·yr^-1^. How nitrogen deposition affects the NAEs of mixed litter decomposition on basic soil chemical and enzyme properties was investigated, and whether increasing litter diversity helps mitigate its potential negative effects was analyzed. The results can provide data support for a deeper understanding of the direct and indirect combined effects of atmospheric nitrogen deposition on soil properties in ecosystems and for formulating corresponding silvicultural management strategies. This study hypothesized that (1) nitrogen deposition will significantly alter the NAEs of mixed litters on basic soil biochemical properties and (2) The increase in litter diversity and the presence of specific species litter in the mixture will mitigate the possible adverse effects of nitrogen deposition.

## 2 Materials and methods

### 2.1 Sampling area

Given the limited influence range of litter addition treatments under field conditions, difficulties in fixing simulated litter layers, and unavoidable random disturbances from nontarget litter and wild animals and plants, this study was conducted via laboratory simulation experiments.

Test samples were collected from the Zhifanggou Watershed in Ansai District, Yan’an City (109°13’–109°16’ E, 36°30’–37°19’ N), which belongs to the hilly and gully region of the Loess Plateau, with an altitude of 1010–1431 m. The climate is a warm-temperate semiarid climate, with a mean annual temperature of 8.8 °C, mean annual precipitation of 549.1 mm, and mean annual evaporation of 1463 mm.

The vegetation type here belongs to the forest-steppe zone, a transitional zone from warm-temperate broad-leaved forests to dry steppes. The native vegetation has undergone severe historical destruction, and the current vegetation is dominated by secondary vegetation such as *R. pseudoacacia*, *Hippophae rhamnoides* and *Caragana korshinskii* plantations, as well as abandoned grasslands. Herbaceous composition varies considerably across regions, with understory or grassland communities dominated by *A. gmelinii*, *C. indicum*, *S. grandis*, *Setaria viridis*, *Heteropappus altaicus*, *S. bungeana*, *Patrinia heterophylla*, *Melica scabrosa*, *Poa sphondylodes* and *Bothriochloa ischaemum*.

### 2.2 Litter and soil sampling and treatment

In late autumn, several 1 m × 1 m quadrats were established in a mature *R. pseudoacacia* (Rp) plantation. Standing dead litter of three dominant understory herbaceous species, including *A. gmelinii* (Ag), *C. indicum* (Ci), and *S. grandis* (Sg), was collected by cutting. After removing the rotten and damaged parts, each type of litter was cut into < 10 cm segments, homogenized, and air-dried. Meanwhile, litter traps were installed in the forest to collect fallen leaves of Rp. To ensure consistent and homogeneous chemical traits in the following experiment, petioles were removed, and the leaves were thoroughly mixed and air-dried.

In addition, subsamples of air-dried litter were oven-dried at 65 °C to constant weight to determine moisture content. All litter mentioned in this study was converted to oven-dried mass hereafter.

The core objective of this study was to explore how the NAEs of litter decomposition on soil properties respond to nitrogen deposition, as well as the differences in such responses of litters with different diversity and compositions. To control variables such as the influence of varying litter ratios and simplify the experiment, this study adopted the methods reported in previous studies on mixed litter decomposition (Wang et al., 2022; Wu et al., 2023), the uneven mixing patterns of litter in natural environments were not simulated. In addition, the number of species in litter mixtures was set to the minimum (2 species) and maximum (4 species) levels among all tested plant materials. Finally, seven litter combinations with equal mass ratios were designed: RpAg, RpCi, RpSg, AgCi, AgSg, CiSg, and RpAgCiSg. Meanwhile, treatments with the four monospecific litters were established to investigate their effects on soil properties, which served as the basic data for calculating the predicted values of the effects induced by mixed litter decomposition. A total of 12 samples were prepared for each monospecific litter and mixed litter group. These samples were used for four levels of nitrogen deposition treatments in the following experiments, with three replicates per treatment. The weight of each litter sample was 10.00 g. For two-species mixtures, each component accounted for 5 g; for the four-species mixture, each component accounted for 2.5 g.

Soil microbial communities and their functional characteristics in forest soils have long adapted to litter inputs dominated by Rp. The home-field advantage may facilitate the decomposition of Rp litter, while its potential effects on the litter of Sg, Ag and Ci — which colonized the understory in the late succession stage — remain unclear. Uniform soil conditions for all litter treatments enabled an accurate assessment of mixed litter decomposition and its feedback on soil properties, and minimized experimental biases caused by soil legacy effects. In addition, the use of external soil (non-forest topsoil) could moderately magnify the combined impacts of (mixed) litter decomposition and nitrogen deposition on soil properties, facilitating experimental detection. Therefore, the test soil was collected from non-forested wasteland over 1 km away from the sampling forest stands.

Specifically, ten 1 m × 1 small quadrats were randomly laid out on the wasteland. After removing surface debris, soils from the 0–5 cm layer were collected. The collected soils were thoroughly mixed and sieved through a 1 mm mesh to remove plant residues and other impurities. The soil saturated water capacity was measured in advance, and soil moisture was adjusted to 50% of this value. This moisture content was close to the actual soil moisture in the local area one day after summer rainfall, which could accelerate litter decomposition and shorten the experiment period.

### 2.3 Litter decomposition incubation and nitrogen deposition treatments

A microcosm for litter decomposition incubation was constructed using a polypropylene plastic box with waterproof and breathable lids. The microcosm had a projected area of 17 cm × 11.5 cm, a height of 4.5 cm and a volume of 750 mL. A total of 200 g of appropriately air-dried soil (converted to oven-dry weight) was spread evenly in each box, followed by a single type or mixed litter sample laid uniformly on the soil surface. The 12 microcosms for each litter treatment were divided into four groups, designated as no nitrogen deposition (N_0_), low nitrogen (N_1_), medium nitrogen (N_2_) and high nitrogen (N_3_) deposition treatments respectively.

Referring to the widely adopted nitrogen deposition levels, actual forms and proportions of deposited nitrogen in relevant studies on the Loess Plateau (Dong et al., 2019; Jing and Wang, 2020), and taking into account the increasing trend of local nitrogen deposition in the future (Dong et al., 2019), four nitrogen deposition rates were determined for the litter-soil microcosms: 0, 4, 8 and 12 g·m^-2^·yr^-1^ for N_0_, N_1_, N_2_ and N_3_ treatments, respectively. At all nitrogen levels, inorganic nitrogen including nitrate nitrogen and ammonium nitrogen each accounted for 35%, while organic nitrogen including glycine and urea each accounted for 15%.

The nitrogen stock solution was prepared by dissolving 3.14 g glycine (NH_2_CH_2_COOH), 1.26 g urea (CO(NH_2_)_2_) and 7.82 g ammonium nitrate (NH_4_NO_3_) in 2 L of distilled water, with the actual nitrogen concentration of 1.9556 g·L^-1^. For the three microcosms of the N_3_ treatment, 10 mL of the stock solution was slowly and evenly sprayed. For the N_2_ and N_1_ treatments, 10 mL of the stock solution diluted 1.5-fold and 3-fold was applied to each of the three microcosms respectively. The three microcosms of the N_0_ treatment were sprayed with 10 mL of sterilized distilled water. The time of nitrogen application was defined as the starting point of decomposition. The incubation lasted for 326 days at a temperature of 20-25 °C. During the experiment, 10 mL of nitrogen solution with corresponding concentration was sprayed into each microcosm every 30 days to maintain the designed nitrogen deposition rate, which was verified by calculating nitrogen concentration of solution, spray volume, projected area and application frequency. The breathable lids remained closed throughout the incubation. Except during nitrogen application, distilled water was added every two weeks based on the weight loss of the microcosms to keep soil moisture stable.

Additionally, blank controls without litter were set for each nitrogen deposition level (three replicates per group) to distinguish the effects of litter decomposition on soil properties.

Upon completion of the decomposition experiment, all residual litter on the soil surface was removed. Each soil sample was air-dried and thoroughly mixed, then sieved through a 1 mm mesh. A portion of the sieved soil was soaked in water to confirm complete separation of litter residues by checking for floating debris. The remaining soil was divided and sieved with 0.149 mm and 0.25 mm sieves for the determination of soil biochemical indices.

### 2.4 Determination of soil and litter properties

Reserved initial samples of each litter type were smashed and sieved through a 0.149 mm sieve. Carbon content was determined using the potassium dichromate external heating method. After digestion with sulfuric acid−hydrogen peroxide, the nitrogen content of the litter was measured with a K−375 automatic Kjeldahl analyzer (Buchi, Switzerland), and the phosphorus content was determined by vanadomolybdate yellow colorimetry. Their lignin and cellulose contents were determined using the acetyl bromide method and sulfuric acid−anthrone colorimetry, respectively (Mao et al., 2015). Their condensed tannin and water−soluble phenol contents were analyzed by vanillin−hydrochloric acid colorimetry and Folin−Ciocalteu colorimetry, respectively (Zhang et al., 2022). Related stoichiometric ratios were calculated accordingly (**Table 1**).

**Table 1:** Initial chemical properties of the tested litters.

Litter	Content (mg g^-1^)							Stoichiometric ratios		
	Carbon	Nitrogen	Phosphorus	Lignin	Cellulose	Condensed tannins	Water-soluble phenol	C/N	C/P	Lignin/N
Rp	439.98 ± 2.08b	20.77 ± 0.71a	1.47 ± 0.06a	159.95 ± 1.89d	68.30 ± 2.74d	1.19 ± 0.03d	2.06 ± 0.03a	21.22 ± 0.64c	300.92 ± 13.80d	7.72 ± 0.34d
Ag	453.75 ± 8.36a	10.90 ± 0.05c	0.66 ± 0.03c	328.64 ± 2.75a	179.77 ± 6.04b	1.27 ± 0.01c	2.08 ± 0.02a	41.61 ± 0.62a	689.40 ± 27.69b	30.14 ± 0.35a
Ci	450.67 ± 2.50ab	10.65 ± 0.03c	0.39 ± 0.01d	288.26 ± 1.49b	146.46 ± 10.77c	1.48 ± 0.04b	1.14 ± 0.05b	42.33 ± 0.30a	1152.42 ± 34.74a	27.08 ± 0.18b
Sg	459.19 ± 2.40a	15.27 ± 0.17b	0.96 ± 0.02b	239.97 ± 12.63c	212.09 ± 3.70a	2.65 ± 0.02a	1.12 ± 0.04b	30.08 ± 0.46b	477.89 ± 9.96c	15.74 ± 0.98c

Data are represented as the average ± SE, and the different letters in the same column indicate significant differences among the tested litters, *P* < 0.05 (based on Tukey’s honestly significant difference method); Rp, *Robinia pseudoacacia*; Ag, *Artemisia gmelinii*; Ci, *Chrysanthemum indicum*; Sg, *Stipa grandis*; The chemical properties of mixed litter can be calculated based on those of monospecific litter and thus were not listed here.

Soil ammonium nitrogen and nitrate nitrogen contents were determined using potassium chloride extraction-indophenol blue colorimetry and ultraviolet spectrophotometry, respectively. The available phosphorus content was measured by sodium carbonate extraction- and phosphomolybdenum blue colorimetry. Soil pH was determined using the glass electrode method at a soil-to-water ratio of 2.5:1 (Bao, 2000). The activities of soil urease (URE), sucrase (SUC), alkaline phosphatase (ALP), and catalase (CAT) were assayed using the indophenol blue colorimetry, dinitrosalicylic acid colorimetry, disodium phenyl phosphate colorimetry, and potassium permanganate titration methods, respectively (Guan, 1986). The results were expressed as follows: SUC: mass (mg) of glucose produced per gram of soil per day (d); URE: mass (mg) of amino nitrogen produced per gram of soil per day (d); ALP: mass (mg) of phenol produced per gram of soil per day (d); CAT: volume (mL) of 0.01 N potassium permanganate solution consumed by 2 g of soil within 20 minutes. All colorimetric analyses were performed using an Epoch 2 Microplate Reader (BioTek, USA) or a UV-2600 ultraviolet-visible spectrophotometer (Shimadzu, Japan). Titration analyses were conducted using a Titrette digital bottle-top burette (Brand, Germany).

### 2.5 Data processing and statistical analyses

The theoretically predicted value *P_mix_* of each soil bio or chemical property after mixed litter decomposition under each nitrogen deposition treatment was calculated using Equation 1:

(1) $$P_{mix}=\sum_{i=1}^{n}M_{i}\times p_{i}$$

where *M_i_* is the measured value of the soil properties after decomposition of each monospecific litter under a given nitrogen deposition rate, and *p_i_* is the mass percentage of each litter type in the corresponding mixture.

Subsequently, the effect sizes of NAEs of mixed litter decomposition on soil properties were calculated using Equation 2. A positive value indicates a synergistic effect of mixed litter decomposition on soil properties, while a negative value indicates an antagonistic effect. When the value is not significantly different from 0 (t test, significance level α=0.05), it is recognized as an additive effect.

(2) $$NAEs=(P_{mix}-M_{mix})/M_{mix}\times 100\%$$

Based on the initial chemical traits of monospecific litters and their mass proportions in different mixtures, two chemical diversity indices, namely, functional richness (FRic) and functional dispersion (FDis), were calculated using FDiversity software (Casanoves et al., 2011).

Given that the NAEs of mixed decomposition of the tested litters generally showed a monotonic increase or decrease with increasing nitrogen deposition rate (unpublished data), this study simplified such relationships into linear functions for convenient evaluation. The changes in NAEs of each mixture on each soil property across different nitrogen deposition rates (CNAEs) were fitted using linear regression, and the slope value was used to quantify the CNAEs. A slope value with no significant difference from zero indicated that nitrogen exerted a significant regulatory effect on NAEs. A positive slope suggested that the changes in NAEs tend to performed as synergistic promoting with increasing deposition rates, and negative ones indicated opposite changes.

The larger the absolute value of the slope, the stronger the corresponding effect. Redundancy analysis (RDA) was applied to explore the relationships between the slope values of various soil indices, and the species diversity, chemical diversity and the presence of specific species of corresponding litter mixtures.

The significance level for all analyses was set at α=0.05. All statistical analyses and plotting were conducted using SPSS 23.0 and OriginPro 2021b software.

## 3 Results

### 3.1 Chemical diversity of the tested litter mixtures

**Figure 1:**
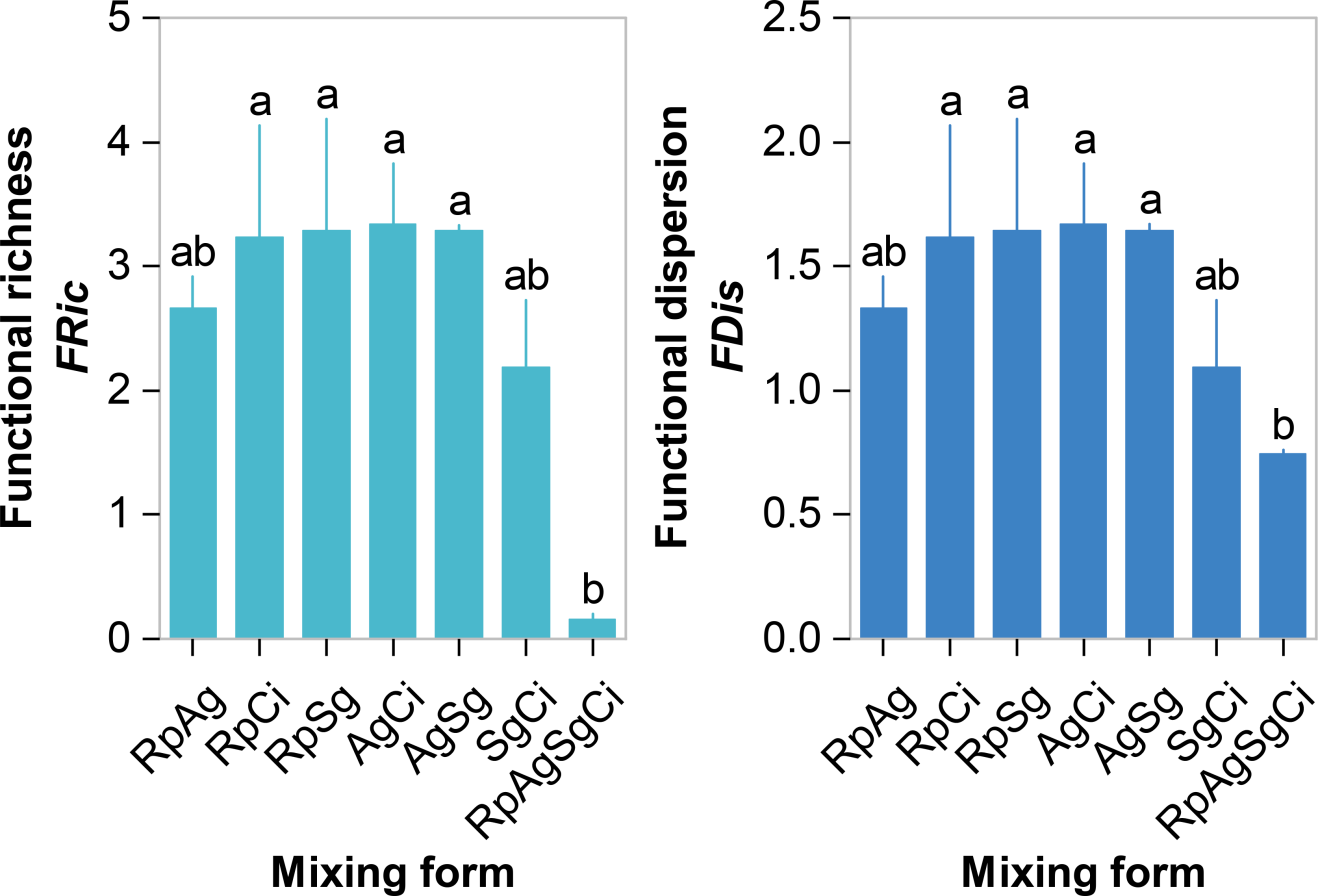
The chemical richness and dispersion of the tested mixed litters. Data are represented as the average ± SE, and the different letters indicate significant differences among litter types, *P* < 0.05 (based on Tukey’s honestly significant difference method). Rp, *Robinia pseudoacacia*; Ag, *Artemisia gmelinii*; Ci, *Chrysanthemum indicum*; Sg, *Stipa grandis*.

The chemical richness and chemical dispersion of the RpCi, RpSg, AgCi, and AgSg litter mixtures were significantly higher than those of the RpAgSgCi mixture (*P* < 0.05). The corresponding indices for the RpAg and SgCi mixtures were intermediate between the two aforementioned mixture types but only showed a tendency of difference (**Figure 1**, *P* > 0.05).

### 3.2 Changes in NAEs on soil chemical properties caused by increasing nitrogen deposition rates

After 326 days of decomposition, the monospecific litter significantly affected the chemical properties of the soil (**Table S1**). Specifically, decomposition of Rp, Ag and Sg litter significantly increased the soil organic carbon content. All litter types significantly reduced the soil nitrate nitrogen content but increased the ammonium nitrogen content. The litter of Rp and Ag increased the soil available phosphorus content, whereas the Ci litter showed the opposite effect. In addition, decomposition of all litter types decreased the soil pH (*P* < 0.05).

After 326 days of decomposition, monospecific litter decomposition significantly increased the contents of soil organic carbon, ammonium nitrogen and available nitrogen under all nitrogen deposition rates, while reducing soil nitrate nitrogen content and soil pH (**Supplementary Table 1**, *P* < 0.05). .

**Figure 2:**
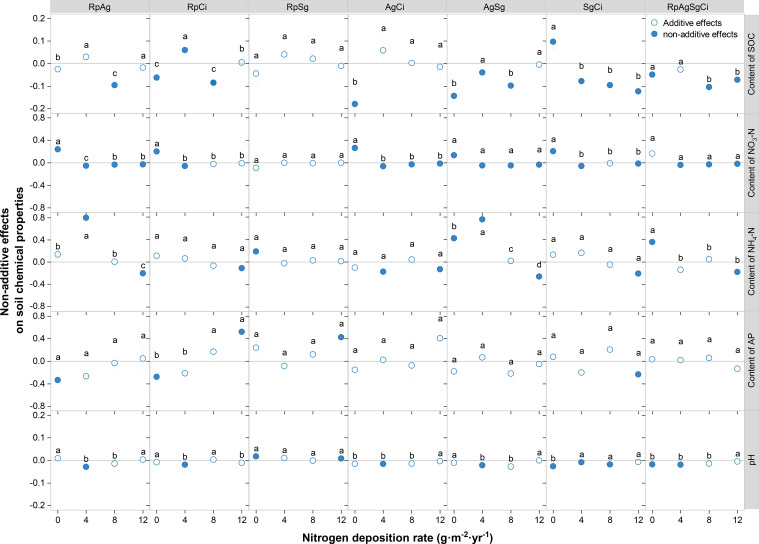
Changes in the nonadditive effects (NAEs) of litter decomposition on soil chemical properties with increasing nitrogen deposition rate. Data are represented as mean value (circles) and SE (error bars), different letters indicate significant differences among the value of NAEs observed under simulated nitrogen deposition treats with different levels (*P* < 0.05, based on Tukey’s honestly significant difference method). The NAEs were calculated based on the data listed in **Supplementary Table 1**.

Under the condition of no nitrogen deposition, mixing of RpCi, AgCi, AgSg and RpAgSgCi antagonistically weakened their effect of replenishing soil organic carbon (*P* < 0.05); while mixing of SgCi litter exhibited synergistic enhancement (*P* < 0.05). Mixing of RpAg, RpCi, AgCi and SgCi litter synergistically enhanced their effect of replenishing soil nitrate nitrogen, and mixing RpSg, AgSg and RpAgSgCi litter synergistically enhanced their effects of replenishing ammonium nitrogen (*P* < 0.05). Mixing of RpAg and RpCi litter antagonistically weakened their effect of replenishing soil available phosphorus (*P* < 0.05). Mixing of RpSg litter antagonistically weakened their effect of acidizing soil, whereas mixing of AgSg, SgCi and RpAgSgCi litter exhibited the opposite effect (**Figure 2**, *P* < 0.05).

However, with increasing nitrogen deposition rates, the antagonistic effects caused by mixing RpCi, AgCi and AgSg litter on organic carbon replenishment tended to be weakened, while the synergistic effect caused by mixing SgCi shifted to significant antagonistic effects (*P* < 0.05). The synergistic or additive effects of nearly all mixing forms on nitrate and ammonium nitrogen replenishment eventually disappeared or converted to significant antagonistic effects (*P* < 0.05). The antagonistic effects caused by the mixing of RpAg and RpCi litter on soil available phosphorus replenishment were significantly weakened and even reversed to significant synergistic effects, whereas the additive effects caused by the mixing of RpSg and SgCi litter gradually shifted to significant synergistic and antagonistic effects, respectively (*P* < 0.05). The additive effects caused by the mixing of RpAg, RpCi and AgCi litter on soil acidification briefly turned into antagonistic effects under low nitrogen deposition rates and then recovered. The antagonistic effects caused by the mixing of AgSg, SgCi and RpAgSgCi litter ultimately disappeared, whereas the synergistic effect caused by the mixing of RpSg litter disappeared temporarily but regained significance under high nitrogen deposition rates (*P* < 0.05).

### 3.3 Changes in NAEs on soil enzyme activities caused by increasing nitrogen deposition rates

Under all nitrogen deposition rates, nearly all monospecific litter decomposition significantly increased the activities of soil sucrase, urease, alkaline phosphatase and catalase (*P* < 0.05, **Supplementary Table 2**). Under the condition of no nitrogen deposition, mixing RpSg, AgSg and SgCi litter antagonistically weakened their effect of stimulating soil sucrase activity (*P* < 0.05). Mixing of RpAgSgCi litter synergistically enhanced their effects of stimulating soil urease activity, while mixing SgCi litter antagonistically weakened their effect of stimulating alkaline phosphatase and catalase activities (*P* < 0.05).

**Figure 3:**
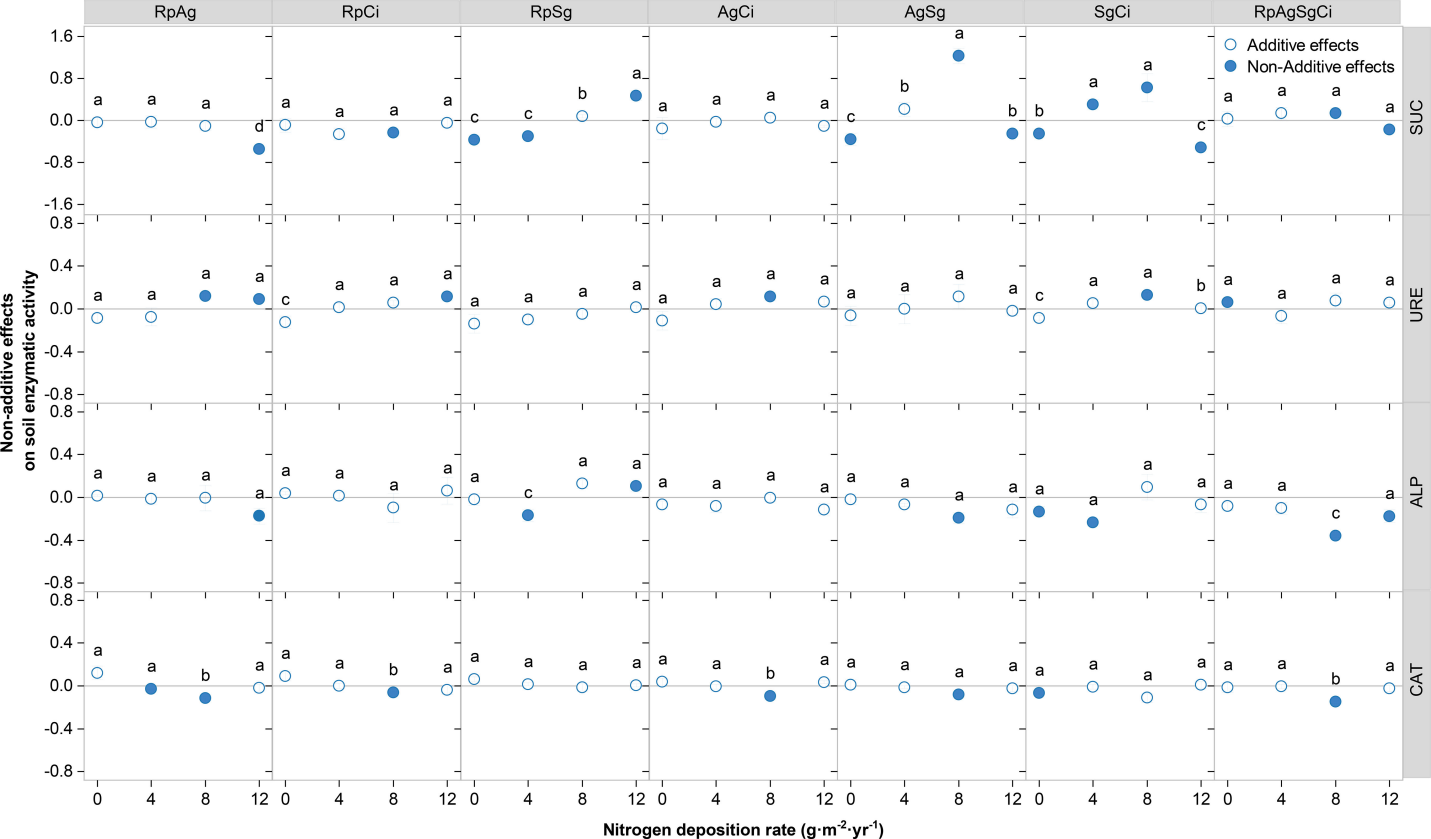
Changes in the nonadditive effects (NAEs) of litter decomposition on soil enzyme activities with increasing nitrogen deposition rate. Data are represented as mean value (circles) and SE (error bars), different letters indicate significant differences among the value of NAEs observed under simulated nitrogen deposition treats with different levels (*P* < 0.05, based on Tukey’s honestly significant difference method). The NAEs were calculated based on the data listed in **Supplementary Table 2**.

However, with increasing nitrogen deposition rates, the additive effects caused by the mixing of RpAg and RpCi litter on sucrase activity gradually shifted to significant antagonism (or recovered afterward), the antagonistic effect caused by the mixing of RpSg litter weakened and turned into significant synergistic enhancement, and the antagonistic or additive effects induced by mixing of AgSg, SgCi and RpAgSgCi litter first shifted toward synergism but then reverted to antagonism (*P* < 0.05). The additive effects caused by the mixing of RpAg, RpCi, AgCi and SgCi litter on urease activity gradually became significant synergistic effects (or recovered afterward), whereas the synergistic effect caused by the mixing of RpAgSgCi litter disappeared completely (*P* < 0.05). The additive effects caused by the mixing of RpAg and RpAgSgCi litter on phosphatase activity shifted toward significant antagonism, the additive effect caused by the mixing of RpSg litter showed the opposite trend, and the antagonistic effect caused by the mixing of SgCi litter weakened and eventually disappeared (*P* < 0.05). The additive effects caused by the mixing of RpAg, RpCi, AgCi, AgSg and RpAgSgCi litter on catalase activity all gradually converted to significant antagonism but then recovered, while the antagonistic effect caused by the mixing of SgCi litter tended to disappear (**Figure 3**, P < 0.05).

### 3.4 How litter diversity and composition affect the response pattern of NAEs to increasing nitrogen deposition rates

**Figure 4:**
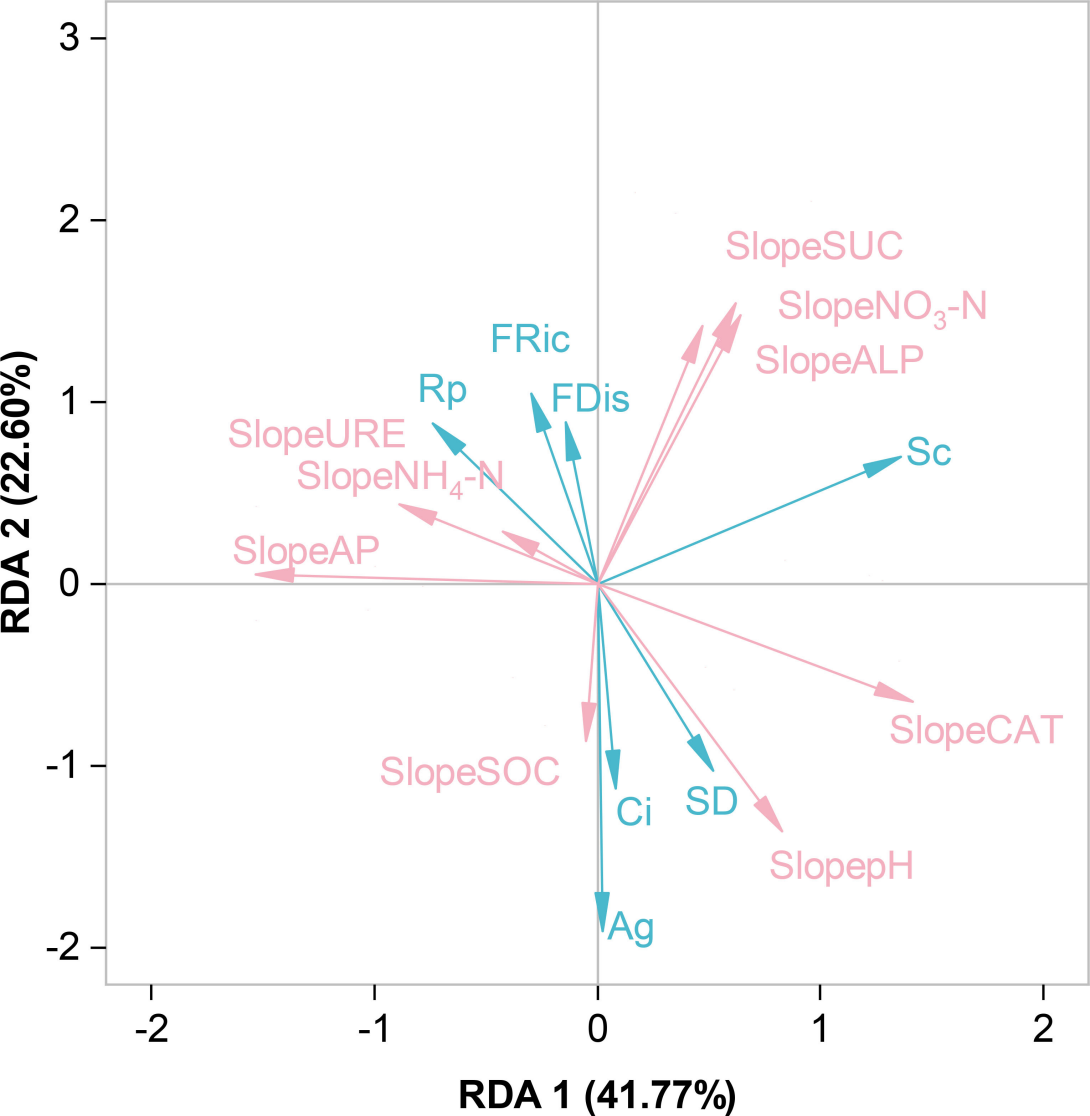
Relationships among litter diversity and composition and the changes in the nonadditive effects (CNAEs) of litter decomposition on soil properties with increasing nitrogen deposition rate. The CNAEs values for a given soil indicator with increasing nitrogen deposition rate were fitted using a linear equation and represented as the slope of the equations. URE, urease activity; SUC, sucrase activity; ALP, alkaline phosphatase activity; CAT, catalase activity; SOC, NO_3_-N, NH_4_-N and AP, contents of soil organic carbon, nitrite nitrogen, ammonium nitrogen and available phosphorus. Rp, *Robinia pseudoacacia*; Ag, *Artemisia gmelinii*; Ci, *Chrysanthemum indicum*; Sg, *Stipa grandis*; SD, litter species diversity; FRic, functional richness; FDis, functional dispersion.

The results of redundancy analysis (RDA, **Figure 4**) indicated that for litter mixtures with high chemical diversity (richness and dispersion) or containing Rp litter, their NAEs on soil carbon and nitrogen supplementation and the activation of soil sucrase, phosphatase and urease tended to shift toward synergistic promotion as nitrogen deposition increased. Meanwhile, the NAEs related to the stimulation of soil catalase activity and soil acidification tended to show antagonistic inhibition. In contrast, litter mixtures with high species diversity and containing Ci and Ag litter caused the opposite results.

## 4 Discussion

### 4.1 The NAEs caused by mixed litter decomposition on soil chemical properties and their changes with increasing nitrogen deposition rates

The results showed that under the condition of no nitrogen deposition, several litter mixture types exerted significant NAEs on soil organic carbon (5/7), available nutrient contents (2/7–4/7) and pH value. In all cases with significant NAEs, the mixing of litter generally tended to weaken the effects of replenishing soil organic carbon and available phosphorus while enhancing the effects of replenishing soil available nitrogen and acidizing soil, which is consistent with the findings of Li et al (Li et al., 2016a; Li et al., 2020). This can be explained by the fact that mixed decomposition may accelerate the release of organic matter and nutrients from litter, leading to a remarkable increase in their accumulation in soil over the same period (Zhou et al., 2023). However, on the other hand, diverse substrates supplied by mixed litter can stimulate the rapid proliferation of soil microorganisms. For instance, Li et al. (2020) reported that when Rp litter decomposed together with litters of *B. platyphylla* and other species, the total amount of culturable bacteria and fungi in the decomposition soil was 3.8 to 9.5 times higher than the predicted value. This is highly likely to accelerate the mineralization and decomposition of native soil organic matter (Zhao et al., 2025b), thus weakening the ability of litter to replenish soil organic carbon. In the phosphorus-deficient environment of the Loess Plateau, this may stimulate microbial phosphorus immobilization and hinder litter-derived phosphorus supplementation to soil. Meanwhile, accelerated litter decomposition may further strengthen its effect on increasing soil available nitrogen. With regard to soil pH, numerous studies have shown that litter decomposition produces acidic intermediates (Zhou et al., 2023), and most organic acids in litter are released in the early stage of decomposition. Therefore, the promotion of litter decomposition by mixing may also be an important reason for the enhanced soil acidification effect (Zhang et al., 2023).

However, a considerable number of litter combinations such as RpAg, RpSg and AgCi did not significantly alter their non-additive effects on soil properties in most cases. Two possible reasons are proposed as follows: (1) The decomposition degree of some litter mixtures was relatively limited. For example, the decomposition rate of the above three combinations was only approximately 45% by the end of the experiment, while that of other mixtures exceeded 60% (unpublished data), thus weakening the impacts of released organic matter and mineralized nutrients on soil properties; (2) Promoting and inhibitory effects of mixed decomposition on soil properties may coexist, and their direct and indirect effects on soil available nutrients (e.g., altering nutrient availability via changes in microbes, enzyme activities, and pH) may offset each other, resulting in apparent additive effects (Zhang et al., 2023).

Consistent with our hypothesis, nitrogen deposition significantly altered the NAEs of mixed litter on soil chemical properties. With increasing nitrogen deposition rates, the antagonistic effects caused by the mixing of some litter on their effects of replenishing soil organic carbon were weakened, while the opposite trend occurred under other mixtures. This may be attributed to the fact that nitrogen addition stimulates microbial growth and facilitates hypha-mediated material transfer between litters, thereby inducing a stronger synergistic effect on mixed litter decomposition (Li et al., 2026). In this study, the synergistic promotion effect on the decomposition rate of the AgCi litter mixture under the N_3_ treatment was significantly higher than that under the N_0_ treatment (*P* < 0.05; NAEs for N_3_ = 22.39%, NAEs for N_0_ = 4.10%, unpublished data), which accelerated the release of more organic debris from litter into the soil. Some debris with high lignin content (such as Ag litter, **Table 1**) may bind to excess nitrogen in the soil and form more stable compounds (Zhang et al., 2020). This consequently results in an apparent trend that high-rate nitrogen deposition improves the capacity of litter to replenish soil organic carbon. In contrast, the synergistic effect on the decomposition rate of the SgCi litter mixture was also significantly greater under N_3_ than under N_0_ (*P* < 0.05; 27.52% vs. 14.22%). However, the NAEs of this mixture on soil organic carbon supplementation decreased markedly at the N_3_ level. The underlying mechanism is that the enhanced synergism induced by nitrogen addition accelerates the release of labile organic matter from mixed litter (Li et al., 2025). This further intensifies the decomposition of native soil organic matter and ultimately leads to a substantial reduction in residual soil organic carbon. It has been reported that the input of soluble sugars can markedly increase soil microbial biomass and the abundance of r-strategic bacteria such as Proteobacteria, triggering an obvious priming effect and consuming native soil organic matter (Zhang et al., 2019).

In contrast to the above phenomenon, exogenous nitrogen addition tended to weaken the synergistic effect caused by litter mixing on the effects of replenishing soil ammonium and nitrate nitrogen. This may be because nitrogen addition accelerates litter decomposition by inducing stronger synergistic effects but indirectly promotes nitrogen immobilization in litter, leading to reduced nitrogen release from litter during the experiment. In addition, under the condition of no nitrogen deposition, mixed decomposition may indeed promote soil microbial growth and enzyme secretion by providing complementary nutrients, thereby enhancing the activation of soil nitrogen (Zhang et al., 2023). However, under relatively high exogenous nitrogen inputs, microbial resource allocation to nitrogen-acquiring enzymes may also decrease significantly (Huang et al., 2021), thus resulting in the opposite effect. For available phosphorus, the NAEs on its replenishment by litter tended to be synergistically enhanced under nitrogen deposition. This may be attributed to the inherent phosphorus deficiency in soils of the Loess Plateau, where the maintenance of phosphorus availability highly depends on litter phosphorus return (Zhang et al., 2019a). Therefore, the enhancement of synergistic effects in mixed litter decomposition by nitrogen deposition promotes phosphorus release of some P-rich litters (Li et al., 2025), making the replenishment of soil available phosphorus more likely to be synergistically enhanced. Finally, the soil acidification effect of litter tended to be antagonistically weakened under high nitrogen deposition rate treatments. This may be because although nitrogen deposition promotes the release of acidic intermediates from mixed litter into the soil by inducing stronger synergistic effects, these intermediates are further degraded under sufficient nitrogen supply, leading to an observed increase in pH, and the specific mechanism remains to be clarified.

### 4.2 The NAEs caused by mixed litter decomposition on soil enzyme activities and their changes with increasing nitrogen deposition rates

In contrast to previous findings (Li et al., 2016a; Li et al., 2020), only a very small number of litter mixture types exerted significant NAEs on soil enzyme activity stimulation under the condition of no nitrogen deposition. The discrepancy may be attributed to the fact that in those studies, litter was applied to soil as small fragments (< 1 mm), which decomposed more rapidly and exerted more direct and sufficient effects on soil microorganisms, nutrient status and pH. Consequently, their mixed decomposition effects had stronger impacts on microbial growth, substrate diversity, release rates of inhibitory metabolites, and soil pH, which are closely related to enzyme activities. In contrast, litter in the present study was placed on the soil surface as intact layers, so the NAEs of mixed decomposition on soil properties were relatively weakened. Notably, the simulated decomposition conditions in this study are closer to the realistic litter–soil interface in natural ecosystems while also minimizing possible inaccuracies in measured soil properties caused by residual litter fragments, as reported in previous investigations (Li et al., 2016a; Li et al., 2020). Future studies are needed to compare the above three experimental approaches to clarify the real effects of litter layers on soil enzyme activities and their changing trends along with regional environmental alterations.

Similar to soil chemical properties, nitrogen deposition only significantly altered the NAEs of mixed litter on the four key soil enzyme activities in approximately 50% of cases. With increasing nitrogen deposition rates, the stimulatory effects of mixed litter on sucrase, phosphatase and catalase activities tended to be antagonistically weakened. This may be because the release of various secondary metabolites is positively correlated with the overall decomposition rate of litter (Zhang et al., 2023). Therefore, the enhancement of the synergistic effect on litter decomposition by nitrogen deposition (such as the decomposition of AgCi mixture mentioned in section 4.1) led to a significant increase in the release of inhibitory secondary metabolites such as phenols (tannins, polyphenolic compounds, etc.) from mixed litter per unit time, allowing them to rapidly accumulate to effective concentrations, thereby shifting the NAEs on soil enzyme activities toward antagonism through direct binding with enzymes (proteins) to inactivate them, inhibiting the growth of microbial groups that secrete related enzymes, and altering soil pH (Chomel et al., 2016). Notably, with increasing nitrogen deposition, the stimulatory effect on soil urease activity tended to be synergistically enhanced, which is consistent with some findings of Li et al (Li et al., 2016a; Li et al., 2020). In those previous studies, the authors suggested that the promotion of litter decomposition by mixing and the consequent improvement of soil properties were the key reasons for the synergistic enhancement of soil enzyme activities (Li et al., 2016a; Li et al., 2020). However, in the present study, with increasing nitrogen deposition, the effects of mixed litter on soil nutrient replenishment—especially on organic carbon and available phosphorus—tended to be antagonistically weakened, which is inconsistent with the above conclusion. Therefore, given that part of the simulated nitrogen deposition in this study was applied in the form of urea, we prefer the explanation that increasing urea input led to substrate accumulation, which induced microorganisms to secrete large amounts of urease for nitrogen acquisition. This phenomenon interfered with the effect of mixed decomposition on soil urease activity, resulting in an apparent trend that litter tended to synergistically promote soil urease activity under high nitrogen deposition rates. The specific mechanism still needs to be further clarified.

### 4.3 Regulation of the response patterns of NAEs to increasing nitrogen deposition by litter diversity and composition

Overall, higher chemical diversity of litter mixtures and the presence of Rp litter drove the NAEs of litter on soil nutrient supplementation and enzyme activities to shift toward synergistic effects with increasing nitrogen deposition rates. By contrast, the rise in species diversity and the presence of Ci and Ag litter exerted the opposite impacts.

The above phenomena can be explained as follows. Litter mixtures with higher chemical diversity provide more diverse and complementary substrates for microorganisms, balancing their demands for various carbon sources and nutrients and increasing the diversity of decomposers (Santonja et al., 2017). Such redundancy at both species and functional levels can substantially enhance the adaptability of decomposers to environmental changes such as nitrogen deposition (Yan et al., 2019). Additionally, previous studies have demonstrated that high-quality litter with high nitrogen and phosphorus contents and a low C:N ratio can increase the relative abundance of copiotrophic microorganisms including Actinobacteria, Proteobacteria and Ascomycota, while significantly reducing the abundance of oligotrophic and nitrogen-inhibition sensitive and Basidiomycota fungi (Dong et al., 2022). In this study, Rp litter was nutrient-rich with low ratios of C:N, C:P and lignin:N (**Table 1**), and it may produce similar effects. Accordingly, litter mixtures with high chemical diversity and Rp litter enable decomposers to better resist the adverse impacts of high-rate nitrogen deposition; instead, sufficient nitrogen supply stimulates microbial growth (Yan et al., 2019). Under the combined action of these factors, the inhibitory effect of nitrogen deposition on decomposers is greatly alleviated or even reversed to a stimulatory effect. This markedly facilitates nutrient exchange among different litters, and the processes of litter decomposition, organic matter and nutrient release and transformation, as well as the consequent improvement of soil biochemical properties, tend to exhibit stronger synergistic promotion.

Conversely, Ci and Ag litters had lower nutrient concentrations and significantly higher contents of lignin and inhibitory secondary metabolites than the other litter types (**Table 1**). According to previous studies, during mixed decomposition, such litter types may inhibit decomposer growth via the release of inhibitory compounds and nutrient acquisition from high-quality litter, thus tending to induce antagonistic effects and weaken the improvement of soil properties by litter (Zhao et al., 2025a). Secondly, their presence usually increases the relative abundance of Basidiomycota in the decomposer communities of mixed litter (Dong et al., 2022). Accordingly, the growth of decomposers is more vulnerable to significant inhibition under nitrogen deposition, which weakens the synergistic effects of mixed decomposition on the transformation and release of litter materials. In addition, due to their high lignin content, these litter types may more readily bind with exogenous nitrogen even under low nitrogen deposition rates, restricting their own decomposition and further reducing nutrient and organic matter return to soil (Peng et al., 2022). Even if overall mixed decomposition is not inhibited but is instead accelerated by nitrogen addition, the accelerated release of inhibitory secondary metabolites may significantly suppress microbial growth and nutrient transformation functions in soil (Chomel et al., 2016), thereby shifting the NAEs of mixed litter on soil properties toward antagonism under high nitrogen deposition rates. Certainly, further examinations of the community composition and functional characteristics of both litter and soil microbes under each treatment are needed in future studies to verify the above speculations. Notably, the effects of litter species diversity were completely opposite to those of chemical diversity in this study, which is consistent with previous findings (Meier and Bowman, 2008). A possible explanation is that the plant species selected in this study had similar traits. The newly added litter types showed intermediate chemical properties, resulting in lower values of FRic and FDis. Furthermore, some studies have indicated that an increase in species number cannot fully reflect variations in the chemical characteristics (or microbial availability) of litter mixtures. Therefore, we suggest that interpreting the mechanisms based on chemical diversity and the presence of specific species is more reasonable.

### 4.4 Limitations and future research perspectives

1. This study focused on the core scientific issue regarding how litter diversity and the presence of specific species regulate the responses of NAEs of mixed litter decomposition on soil properties to nitrogen deposition. For this purpose, equal mass mixing, a commonly adopted method, was used to eliminate the interference of variable litter mixing ratios under natural conditions (Wang et al., 2022; Wu et al., 2023). In addition, as the shaded environment of mature Rp plantations, the understory dominant species are limited and usually belong to resource-conservative or opportunistic species, generally had relatively high concentrations of carbon, lignin and other secondary metabolites but low nitrogen and phosphorus contents (Bai et al., 2024), such as the selected Sg, Ag and Ci. Furthermore, these species grow in scattered clumps under the canopy, and multi-species litter mixtures are rarely observed in the field. Therefore, this study mainly focused on two-species mixtures and the extreme four-species mixtures. Certainly, although the above settings conform to the actual field conditions and simplify the experimental design, they unavoidably collectively lead to relatively similar chemical diversity (richness and dispersion) among the tested litter mixtures. Consequently, this study failed to fully clarify how different mixing ratios and large-gradient variations in species and chemical diversity shape the community and functional traits of colonizing decomposers, mediate the generation of NAEs, and alter the responses of NAEs to increasing nitrogen deposition. Further studies should incorporate more plant species and establish a wider range of mixing ratios that mimic natural conditions to verify and extend the present findings.
2. In hilly areas, nitrogen may continuously migrate in field plots with simulated nitrogen deposition, resulting in uneven nitrogen distribution and altered nitrogen concentrations. Meanwhile, commonly used litter decomposition bags generally cover an area of only ~10×15 cm^2^, and only quite limited soil can be affected by litter decomposition when using litterbag decomposition method. Therefore, the effects of mixed litter decomposition on soil properties might strongly disturbed by uncontrollable factors such as rainfall leaching, soil erosion, and random invasion of wild animals and plants in filed experiment. To exclude these disturbances, a well-controlled laboratory simulation experiment with uniformly distributed treatments and influencing factors was conducted in this study. Admittedly, the laboratory results cannot fully represent the *in-situ* processes. For future research, large-area litter placement experiments can be carried out at long-term nitrogen deposition monitoring sites with increased plots and sample replicates. Statistical approaches such as generalized linear mixed models can be applied to control and separate random effects, so as to further improve the reliability of research conclusions.
3. This study mainly explored the cumulative effects of NAEs of litter decomposition on soil properties and the modification of these NAEs by nitrogen deposition during the period between litter formation and the emergence of new litter. Litter decomposition and its effects on soil properties, such as the alternating processes of nutrient release and accumulation of soil available nutrients, determine nutrient supply for plant growth in the subsequent year. Thus, it is necessary to further investigate the dynamics of these effects across different plant growth stages. Furthermore, the composition, structure and functional traits of soil microbial communities were not analyzed in this study. Future research should combine high-throughput sequencing technology to clarify the variations in the composition, diversity and functions of fungi and bacteria in the litter layer and soil under altered nitrogen deposition and litter diversity, so as to reveal the underlying microbial mechanisms.

## 5 Conclusions

Nitrogen deposition significantly interfered with the non-additive effects of mixed litter decomposition on soil chemical and biological properties. Higher chemical diversity (richness and dispersion) and the presence of high-quality litter such as *R. pseudoacacia* litter facilitate the shift of non-additive effects of mixed litter on soil properties under nitrogen deposition toward synergistic promotion. By contrast, an increase in the number of litter species and the presence of low-quality litter including *C. indicum* and *A. gmelinii* produce the opposite effects.

## Data Availability

The original contributions presented in the study are included in the article/**Supplementary Material**. Further inquiries can be directed to the corresponding author.
