# Supplementary material for: Nitrogen deposition significantly alters the nonadditive effects of mixed litter decomposition on soil bio and chemical properties
Source: Front Plant Sci. 2026 Jul 7;17:1865577. doi: 10.3389/fpls.2026.1865577 (PMC13385123; doi:10.3389/fpls.2026.1865577)
Supplement: Supplementary file 1 [file Table1.docx]

Nitrogen deposition significantly alters the nonadditive effects of mixed litter decomposition on soil bio and chemical properties

**Supplementary materials**

**TABLE S1** Soil chemical properties in the litter treatments. Data are represented as the average ± SE. Different letters in the same column for monospecific litter treatments indicate significant differences among treatments, *P*<0.05 (based on Tukey's honestly significant difference method); * indicates significant differences between the predicted and observed values for each mixed litter treatment under given nitrogen deposition rates, *P*<0.05; Rp: *Robinia pseudoacacia*, Ag: *Artemisia gmelinii*, Ci: *Chrysanthemum indicum*, Sg: *Stipa grandis*, CK: soil without litter treatment. N_0_, N_1_, N_2_ and N_3_ indicate that the rates of nitrogen deposition are 0, 4, 8 and 12 g·m^-2^·yr^-1^, respectively.

| **Treatments** | | **Contents (mg·g^-1^)** | | | | **pH** |
| --- | --- | --- | --- | --- | --- | --- |
| **Nitrogen treatment** | **Litter type** | **Organic carbon (SOC)** | **Nitrate N (NO_3_-N)** | **Ammonium N (NH_4_-N)** | **Available P (AP)** |  |
| N_0_ | CK | 3.93±0.09b | 120.06±2.55a | 2.76±0.36d | 5.60±0.36b | 8.20±0.01a |
|  | Monospecific | | | | | |
|  | Rp | 4.72±0.15a | 101.34±0.47b | 6.58±0.27b | 8.01±1.08a | 7.46±0.01d |
|  | Ag | 4.50±0.07a | 50.65±2.321d | 6.98±0.46b | 7.39±0.14a | 7.69±0.07cd |
|  | Ci | 3.90±0.08b | 65.05±1.27c | 11.84±0.40a | 3.31±0.21c | 7.75±0.01bc |
|  | Sg | 4.74±0.09a | 67.53±4.88c | 5.06±0.28c | 5.85±0.21b | 7.82±0.02b |
|  | Observed | | | | | |
|  | RpAg | 4.50±0.09 | 94.18±2.22* | 7.67±0.18 | 5.08±0.23* | 7.65±0.03 |
|  | RpCi | 4.04±0.05* | 100.00±2.19* | 10.22±0.31 | 4.08±0.20* | 7.54±0.04 |
|  | RpSg | 4.52±0.25 | 76.35±2.42 | 6.89±0.24* | 8.47±0.62 | 7.77±0.03* |
|  | AgCi | 3.44±0.04* | 72.85±0.51* | 8.41±0.45 | 4.54±0.41 | 7.60±0.05 |
|  | AgSg | 3.96±0.10* | 66.63±7.09* | 8.56±0.72* | 5.47±0.96 | 7.67±0.01 |
|  | SgCi | 4.73±0.07* | 79.64±2.77* | 9.55±0.99 | 4.93±0.77 | 7.58±0.02* |
|  | RpAgSgCi | 4.25±0.07* | 82.42±9.26 | 10.29±0.76* | 6.31±0.76 | 7.55±0.01* |
|  | Predicted | | | | | |
|  | RpAg | 4.61±0.05 | 76.00±1.33 | 6.78±0.34 | 7.70±0.61 | 7.58±0.04 |
|  | RpCi | 4.31±0.08 | 83.20±0.49 | 9.21±0.30 | 5.66±0.44 | 7.60±0.01 |
|  | RpSg | 4.73±0.06 | 84.44±2.58 | 5.82±0.09 | 6.93±0.60 | 7.64±0.00 |
|  | AgCi | 4.20±0.04 | 57.85±1.31 | 9.41±0.43 | 5.35±0.04 | 7.72±0.03 |
|  | AgSg | 4.62±0.06 | 59.09±2.48 | 6.02±0.09 | 6.62±0.15 | 7.76±0.03 |
|  | SgCi | 4.32±0.08 | 66.29±1.83 | 8.45±0.07 | 4.58±0.10 | 7.79±0.01 |
|  | RpAgSgCi | 4.47±0.03 | 71.14±1.04 | 7.62±0.20 | 6.14±0.28 | 7.68±0.02 |
| N_1_ | CK | 4.19±0.03b | 168.44±7.98 a | 5.28±0.29 c | 5.15±0.30 b | 8.11±0.01 a |
|  | Monospecific | | | | | |
|  | Rp | 4.66±0.02a | 115.55±0.98 b | 7.86±0.45 a | 8.09±0.39 a | 7.53±0.02 b |
|  | Ag | 4.55±0.10a | 111.55±0.90 b | 7.16±0.39 b | 7.93±0.73 a | 7.56±0.04 b |
|  | Ci | 4.34±0.13ab | 113.79±0.53 b | 10.84±1.55 a | 5.62±0.47 b | 7.38±0.00 c |
|  | Sg | 4.57±0.11a | 111.50±0.49 b | 8.08±0.38 a | 6.13±0.34 a | 7.53±0.04 b |
|  | Observed | | | | | |
|  | RpAg | 4.74±0.05 | 107.41±0.38 | 11.45±2.42 | 5.78±0.67 | 7.33±0.01 |
|  | RpCi | 4.77±0.03 | 108.12±0.45 | 10.53±1.92 | 5.39±0.80 | 7.31±0.01 |
|  | RpSg | 4.80±0.08 | 113.58±0.50 | 7.75±0.44 | 6.47±0.23 | 7.61±0.08 |
|  | AgCi | 4.69±0.11 | 105.66±0.37 | 8.01±0.69 | 6.93±0.23 | 7.35±0.01 |
|  | AgSg | 4.38±0.04 | 106.08±0.10 | 13.30±0.48 | 7.50±0.71 | 7.39±0.00 |
|  | SgCi | 4.11±0.03 | 106.00±0.14 | 11.75±0.76 | 4.70±0.15 | 7.39±0.02 |
|  | RpAgSgCi | 4.41±0.05 | 108.67±0.17 | 7.59±0.62 | 7.09±0.20 | 7.36±0.00 |
|  | Predicted | | | | | |
|  | RpAg | 4.60±0.04 | 113.55±0.61 | 7.51±0.36 | 8.01±0.49 | 7.54±0.02 |
|  | RpCi | 4.50±0.03 | 114.67±0.73 | 9.35±0.59 | 6.85±0.16 | 7.45±0.01 |
|  | RpSg | 4.62±0.11 | 113.52±0.73 | 7.97±0.35 | 7.11±0.34 | 7.53±0.02 |
|  | AgCi | 4.44±0.15 | 112.67±0.35 | 9.00±0.62 | 6.78±0.17 | 7.47±0.02 |
|  | AgSg | 4.56±0.03 | 111.53±0.43 | 7.62±0.39 | 7.03±0.53 | 7.55±0.04 |
|  | SgCi | 4.46±0.04 | 112.65±0.51 | 9.46±0.63 | 5.88±0.07 | 7.46±0.02 |
|  | RpAgSgCi | 4.53±0.02 | 113.10±0.46 | 8.49±0.14 | 6.94±0.21 | 7.50±0.02 |
| N_2_ | CK | 3.81±0.04d | 174.36±7.31a | 6.25±0.32c | 5.14±0.07c | 7.99±0.01a |
|  | Monospecific | | | | | |
|  | Rp | 4.30±0.11a | 119.50±0.90b | 8.29±0.33b | 7.39±0.70a | 7.25±0.04c |
|  | Ag | 4.45±0.07a | 117.69±0.07b | 8.62±0.62b | 6.70±0.74b | 7.47±0.05b |
|  | Ci | 4.07±0.09c | 111.26±1.31c | 7.98±0.23bc | 5.08±1.27c | 7.31±0.02c |
|  | Sg | 4.29±0.05b | 117.29±0.39b | 9.77±0.33a | 7.31±0.28a | 7.35±0.06c |
|  | Observed | | | | | |
|  | RpAg | 3.96±0.02 | 114.71±0.08* | 8.46±0.05* | 6.78±0.73 | 7.25±0.02* |
|  | RpCi | 3.83±0.05* | 112.68±1.00* | 7.55±0.28 | 7.01±0.85 | 7.31±0.01* |
|  | RpSg | 4.39±0.06 | 117.17±0.68 | 9.27±0.25 | 8.09±1.06 | 7.30±0.03 |
|  | AgCi | 4.27±0.06 | 111.17±0.34* | 8.65±0.72* | 5.47±1.27 | 7.28±0.01* |
|  | AgSg | 3.94±0.02* | 112.11±0.44* | 9.36±0.36* | 5.47±0.73 | 7.21±0.01* |
|  | SgCi | 3.78±0.05* | 113.42±0.25* | 8.46±0.23 | 7.24±0.15 | 7.20±0.02* |
|  | RpAgSgCi | 3.83±0.07 | 113.04±0.43* | 9.12±0.77 | 7.01±0.54 | 7.26±0.02* |
|  | Predicted | | | | | |
|  | RpAg | 4.38±0.08 | 118.60±0.42 | 8.45±0.41 | 7.05±0.31 | 7.36±0.04 |
|  | RpCi | 4.19±0.10 | 115.38±1.11 | 8.13±0.26 | 6.24±0.84 | 7.28±0.01 |
|  | RpSg | 4.30±0.03 | 118.40±0.27 | 9.03±0.04 | 7.35±0.39 | 7.30±0.04 |
|  | AgCi | 4.26±0.08 | 114.48±0.63 | 8.30±0.42 | 5.89±0.31 | 7.39±0.02 |
|  | AgSg | 4.37±0.04 | 117.49±0.23 | 9.20±0.24 | 7.01±0.27 | 7.41±0.06 |
|  | SgCi | 4.18±0.04 | 114.28±0.48 | 8.88±0.07 | 6.20±0.77 | 7.33±0.03 |
|  | RpAgSgCi | 4.28±0.05 | 116.44±0.45 | 8.66±0.19 | 6.62±0.29 | 7.35±0.03 |
| CK | | 3.77±0.04b | 172.57±4.67a | 6.34±0.11b | 4.86±0.22c | 7.92±0.01a |
| N_3_ | Monospecific | | | | | |
|  | Rp | 4.27±0.11a | 118.28±0.20b | 9.72±0.52a | 6.39±0.66a | 7.25±0.03b |
|  | Ag | 4.38±0.05a | 118.53±0.21b | 9.88±0.06a | 6.52±0.72a | 7.18±0.03c |
|  | Ci | 4.28±0.01a | 112.90±0.44c | 8.53±0.64a | 4.70±0.43c | 7.31±0.03b |
|  | Sg | 4.41±0.08a | 119.17±0.20b | 10.17±0.37a | 5.62±0.34b | 7.24±0.04b |
|  | Observed | | | | | |
|  | RpAg | 4.25±0.10 | 114.90±0.11* | 7.78±0.07* | 6.47±0.71 | 7.24±0.02 |
|  | RpCi | 4.29±0.03 | 114.68±0.08 | 8.12±0.21* | 8.32±0.53* | 7.20±0.01 |
|  | RpSg | 4.29±0.08 | 118.81±0.53 | 10.11±0.34 | 8.52±0.45* | 7.30±0.01* |
|  | AgCi | 4.27±0.36 | 113.99±0.17* | 8.01±0.25* | 7.70±0.67 | 7.22±0.03 |
|  | AgSg | 4.37±0.11 | 115.12±0.78* | 7.43±0.18* | 5.70±0.28 | 7.21±0.03 |
|  | SgCi | 3.81±0.05* | 114.43±0.34* | 7.34±0.45* | 3.93±0.23* | 7.23±0.01 |
|  | RpAgSgCi | 4.02±0.07* | 114.70±0.46* | 7.89±0.13* | 5.08±0.80* | 7.21±0.02 |
|  | Predicted | | | | | |
|  | RpAg | 4.32±0.05 | 118.40±0.19 | 9.80±0.27 | 6.46±0.69 | 7.21±0.01 |
|  | RpCi | 4.27±0.06 | 115.59±0.16 | 9.13±0.23 | 5.54±0.50 | 7.28±0.01 |
|  | RpSg | 4.34±0.10 | 118.73±0.07 | 9.95±0.23 | 6.01±0.47 | 7.25±0.01 |
|  | AgCi | 4.33±0.02 | 115.71±0.23 | 9.20±0.29 | 5.61±0.51 | 7.24±0.02 |
|  | AgSg | 4.40±0.03 | 118.85±0.01 | 10.02±0.15 | 6.07±0.51 | 7.21±0.03 |
|  | SgCi | 4.34±0.05 | 116.04±0.25 | 9.35±0.50 | 5.16±0.28 | 7.28±0.03 |
|  | RpAgSgCi | 4.33±0.05 | 117.22±0.08 | 9.57±0.18 | 5.81±0.48 | 7.24±0.01 |

**TABLE S2** Soil enzyme activities in the litter treatments. Data are represented as the average ± SE. Different letters in the same column for monospecific litter treatments indicate significant differences among treatments, *P*<0.05 (based on Tukey's honestly significant difference method); * indicates significant differences between the predicted and observed values for each mixed litter treatment under given nitrogen deposition rates, *P*<0.05; Rp: *Robinia pseudoacacia*, Ag: *Artemisia gmelinii*, Ci: *Chrysanthemum indicum*, Sg: *Stipa grandis*, CK: soil without litter treatment. N_0_, N_1_, N_2_ and N_3_ indicate that the rates of nitrogen deposition are 0, 4, 8 and 12 g·m^-2^·yr^-1^, respectively.

| **Nitrogen treatment** | **Litter type** | **Sucrase**  **(mg glucose·g^-1^·d^-1^)** | **Urease**  **(mg NH_2_-N·g^-1^·d^-1^)** | **Alkline phosphatase**  **(mg phenol·g^-1^·d^-1^)** | **Catalase**  **(mL 0.01 N KMnO_4_·2 g^-1^·20 min^-1^)** |
| --- | --- | --- | --- | --- | --- |
| N_0_ | CK | 38.79±1.79d | 2.12±0.12c | 24.95±1.36b | 2.57±0.15b |
|  | Monospecific | | | | |
|  | Rp | 269.41±25.15a | 7.87±0.12a | 41.83±1.24a | 6.65±0.43a |
|  | Ag | 79.26±5.48c | 6.81±0.17b | 38.10±3.11a | 6.89±0.41a |
|  | Ci | 77.70±0.03c | 6.63±0.16b | 41.21±2.15a | 7.60±0.04a |
|  | Sg | 122.3±4.89b | 6.82±0.35b | 42.45±0.62a | 7.63±0.08a |
|  | Observed | | | | |
|  | RpAg | 168.47±20.83 | 6.72±0.33 | 40.59±1.64 | 7.54±0.02 |
|  | RpCi | 154.38±10.87 | 6.38±0.50 | 43.07±1.08 | 7.76±0.09 |
|  | RpSg | 122.30±1.36* | 6.30±0.56 | 41.21±2.15 | 7.57±0.11 |
|  | AgCi | 66.74±16.34 | 5.96±0.54 | 36.86±1.64 | 7.49±0.07 |
|  | AgSg | 64.37±12.13* | 6.35±0.50 | 39.34±0.00 | 7.32±0.10 |
|  | SgCi | 74.57±3.41* | 6.15±0.34 | 36.24±1.24* | 7.12±0.04* |
|  | RpAgSgCi | 138.59±13.31 | 7.46±0.04* | 37.48±1.86 | 7.06±0.18 |
|  | Predicted | | | | |
|  | RpAg | 174.33±15.31 | 7.34±0.04 | 39.97±1.35 | 6.77±0.40 |
|  | RpCi | 173.55±12.57 | 7.25±0.12 | 41.52±0.62 | 7.13±0.23 |
|  | RpSg | 195.85±12.32 | 7.35±0.23 | 42.14±0.54 | 7.14±0.25 |
|  | AgCi | 78.48±2.74 | 6.72±0.04 | 39.65±2.54 | 7.25±0.21 |
|  | AgSg | 100.78±3.41 | 6.82±0.13 | 40.28±1.86 | 7.26±0.25 |
|  | SgCi | 100.00±2.44 | 6.73±0.21 | 41.83±1.35 | 7.62±0.04 |
|  | RpAgSgCi | 137.17±7.52 | 7.03±0.09 | 40.90±1.24 | 7.19±0.22 |
| N_1_ | CK | 35.26±2.06 d | 2.24±0.13 c | 28.53±1.34 b | 3.18±0.37 c |
|  | Monospecific | | | | |
|  | Rp | 239.67±14.28 a | 8.62±0.04 a | 43.70±1.24 a | 7.39±0.12 a |
|  | Ag | 147.78±1.07 b | 6.99±0.40 b | 46.18±1.24 a | 7.45±0.04 a |
|  | Ci | 108.53±2.52 c | 7.82±0.15 a | 41.21±1.08 a | 6.75±0.20 b |
|  | Sg | 94.13±2.35 c | 6.82±0.47 b | 43.69±2.24 a | 7.32±0.12 a |
|  | Observed | | | | |
|  | RpAg | 186.46±16.84 | 7.24±0.81 | 44.32±2.24 | 7.19±0.04* |
|  | RpCi | 128.56±19.47 | 8.34±0.27 | 43.07±1.08 | 7.06±0.10 |
|  | RpSg | 116.04±0.78* | 6.93±0.18 | 36.24±1.64* | 7.46±0.05 |
|  | AgCi | 124.65±9.49 | 7.72±0.15 | 39.97±1.64 | 7.05±0.19 |
|  | AgSg | 146.62±15.25 | 6.86±0.80 | 41.83±1.24 | 7.28±0.07 |
|  | SgCi | 132.33±9.73* | 7.70±0.21 | 32.51±0.62* | 6.98±0.01 |
|  | RpAgSgCi | 166.59±15.05 | 7.05±0.59 | 39.17±2.16 | 7.19±0.08 |
|  | Predicted | | | | |
|  | RpAg | 193.72±6.62 | 7.80±0.18 | 44.94±0.00 | 7.42±0.04 |
|  | RpCi | 174.10±7.01 | 8.22±0.07 | 42.45±0.82 | 7.07±0.10 |
|  | RpSg | 166.90±7.33 | 7.72±0.25 | 43.69±0.82 | 7.36±0.06 |
|  | AgCi | 128.15±1.34 | 7.41±0.25 | 43.69±0.82 | 7.10±0.10 |
|  | AgSg | 120.95±1.37 | 6.91±0.19 | 44.94±1.61 | 7.39±0.07 |
|  | SgCi | 101.33±0.16 | 7.32±0.16 | 42.45±0.82 | 7.04±0.16 |
|  | RpAgSgCi | 147.53±3.24 | 7.56±0.07 | 43.69±0.41 | 7.23±0.07 |
| N_2_ | CK | 39.49±2.49d | 2.85±0.09c | 25.18±2.47c | 3.50±0.38c |
|  | Monospecific | | | | |
|  | Rp | 229.50±6.26a | 7.52±0.03a | 36.24±2.71b | 7.48±0.14a |
|  | Ag | 108.22±1.35c | 7.36±0.48a | 43.07±2.15a | 7.43±0.15a |
|  | Ci | 145.46±16.05b | 7.60±0.29a | 27.36±1.34c | 7.05±0.14b |
|  | Sg | 47.79±3.83d | 6.80±0.47b | 36.86±0.62b | 7.34±0.10a |
|  | Observed | | | | |
|  | RpAg | 150.94±16.49 | 8.31±0.14* | 39.00±2.94 | 6.59±0.04* |
|  | RpCi | 142.64±10.26* | 7.97±0.05 | 28.24±2.79 | 6.82±0.04* |
|  | RpSg | 149.69±4.76 | 6.80±0.13 | 41.21±1.86 | 7.30±0.19 |
|  | AgCi | 132.16±7.29 | 8.34±0.14* | 34.99±2.24 | 6.56±0.17* |
|  | AgSg | 174.73±15.71* | 7.85±0.57 | 32.11±0.89* | 6.79±0.13 |
|  | SgCi | 152.82±16.26* | 8.13±0.18* | 34.99±3.29 | 6.40±0.30* |
|  | RpAgSgCi | 150.47±5.18* | 7.89±0.41 | 22.92±0.51* | 6.23±0.05* |
|  | Predicted | | | | |
|  | RpAg | 168.86±2.57 | 7.44±0.23 | 39.65±2.18 | 7.46±0.12 |
|  | RpCi | 187.48±6.16 | 7.56±0.15 | 31.80±2.00 | 7.27±0.07 |
|  | RpSg | 138.65±1.22 | 7.16±0.23 | 36.55±1.42 | 7.41±0.05 |
|  | AgCi | 126.84±8.24 | 7.48±0.18 | 35.22±1.66 | 7.24±0.13 |
|  | AgSg | 78.00±2.53 | 7.08±0.41 | 39.97±1.35 | 7.39±0.04 |
|  | SgCi | 96.63±9.45 | 7.20±0.09 | 32.11±0.84 | 7.20±0.07 |
|  | RpAgSgCi | 132.74±3.64 | 7.32±0.14 | 35.88±1.50 | 7.33±0.05 |
| N_3_ | CK | 34.36±5.97e | 3.25±0.11b | 27.28±2.15c | 2.46±0.18b |
|  | Monospecific | | | | |
|  | Rp | 173.94±4.89a | 7.94±0.14a | 41.21±2.15a | 6.95±0.09a |
|  | Ag | 111.35±1.56c | 7.89±0.27a | 40.70±1.19a | 6.34±0.21a |
|  | Ci | 144.21±7.46b | 8.25±0.33a | 37.08±1.43a | 6.58±0.05a |
|  | Sg | 84.57±4.93d | 8.16±0.16a | 31.27±0.62b | 6.43±0.06a |
|  | Observed | | | | |
|  | RpAg | 64.16±4.45* | 8.61±0.08* | 33.69±2.11* | 6.52±0.13 |
|  | RpCi | 150.47±12.20 | 9.00±0.13* | 41.21±3.23 | 6.49±0.25 |
|  | RpSg | 189.59±5.90* | 8.17±0.19 | 39.97±0.62* | 6.72±0.07 |
|  | AgCi | 113.69±5.48 | 8.61±0.10 | 34.37±1.24 | 6.69±0.04 |
|  | AgSg | 73.32±3.39* | 7.87±0.35 | 36.24±1.64 | 6.22±0.04 |
|  | SgCi | 54.37±3.12* | 8.25±0.23 | 31.89±2.85 | 6.57±0.24 |
|  | RpAgSgCi | 105.63±2.51* | 8.53±0.09 | 30.87±1.48* | 6.41±0.19 |
|  | Predicted | | | | |
|  | RpAg | 142.64±2.38 | 7.92±0.17 | 40.96±1.19 | 6.65±0.07 |
|  | RpCi | 159.08±5.64 | 8.10±0.23 | 39.15±1.62 | 6.77±0.05 |
|  | RpSg | 129.26±4.29 | 8.05±0.11 | 36.24±1.12 | 6.69±0.04 |
|  | AgCi | 127.78±3.06 | 8.08±0.24 | 38.89±1.22 | 6.46±0.10 |
|  | AgSg | 97.96±3.06 | 8.03±0.21 | 35.98±0.29 | 6.39±0.13 |
|  | SgCi | 114.39±3.82 | 8.21±0.19 | 34.17±0.51 | 6.51±0.02 |
|  | RpAgSgCi | 128.52±3.00 | 8.06±0.17 | 37.56±0.85 | 6.58±0.04 |
